# The lactic dehydrogenase-to-albumin ratio predicts acute kidney injury in patients with intracerebral hemorrhage: a multicenter cohort study

**DOI:** 10.3389/fneur.2025.1606881

**Published:** 2025-12-18

**Authors:** Taotao Dong, Yangchun Xiao, Xiang Yuan, Peng Wang, Chao You, Fang Fang, Yu Zhang

**Affiliations:** 1 Center for Evidence Based Medical, Affiliated Hospital and Clinical Medical College of Chengdu University, Chengdu, Sichuan, China; 2 Department of Neurosurgery, Affiliated Hospital and Clinical Medical College of Chengdu University, Chengdu, Sichuan, China; 3 Department of Neurosurgery, West China Hospital, Sichuan University, Chengdu, Sichuan, China

**Keywords:** intracerebral hemorrhage, acute kidney injury, inflammation, biomarker, prognosis

## Abstract

**Background:**

Acute kidney injury (AKI) is a common and serious complication in patients with intracerebral hemorrhage (ICH), contributing to poor clinical outcomes. The lactic dehydrogenase-to-albumin ratio (LAR) has emerged as a promising biomarker combining markers of cellular injury and systemic inflammation. However, its role in predicting AKI in ICH patients remains unexplored.

**Objective:**

This study aims to evaluate the predictive value of LAR for AKI in ICH patients and assess its potential to enhance predictive models for AKI risk.

**Methods:**

We conducted a retrospective, multicenter cohort study involving 6,465 patients with spontaneous ICH from three hospitals in China. LAR was calculated from routine laboratory values (lactic dehydrogenase and albumin), and its association with AKI was analyzed using logistic regression and receiver operating characteristic (ROC) curves. Subgroup analyses were performed to explore heterogeneity in the relationship between LAR and AKI.

**Results:**

Higher LAR values were significantly associated with increased risk of AKI. Patients with LAR >5.61 had a higher odds ratio (OR 2.51, 95% CI 2.13–2.96) for developing AKI compared to those with lower LAR. A dose-response relationship was observed, with progressively higher AKI risk across LAR quartiles. Incorporating LAR into existing predictive models improved the accuracy from 0.73 to 0.76 (*p* < 0.001). Subgroup analysis revealed that age and hematoma volume influenced the strength of the LAR-AKI association.

**Conclusions:**

LAR is a reliable and cost-effective biomarker associated with AKI risk in ICH patients, with significant potential to enhance early detection and risk stratification; however, causality cannot be established due to the retrospective study design. Its incorporation into predictive models improves accuracy and provides a feasible tool for identifying high-risk patients. Further validation and long-term studies are needed to confirm its clinical utility across diverse populations.

## Background

Acute kidney injury (AKI) is a common and severe complication in patients with intracerebral hemorrhage (ICH), significantly worsening clinical outcomes by prolonging hospitalization, increasing the need for renal replacement therapy, and elevating mortality (1, 2). Although the association between ICH and AKI is well-established, early identification of patients at risk for AKI remains a major clinical challenge due to the lack of reliable and practical predictive biomarkers (3–5). Currently, no widely accepted indicator exists that can effectively predict the onset of AKI in critically ill ICH patients, hindering timely intervention and contributing to poor prognoses.

ICH triggers a cascade of complex pathophysiological processes that pre-dispose patients to AKI, including cerebral ischemia, systemic inflammatory response, and hemodynamic instability (6–8). These processes lead to widespread endothelial dysfunction, oxidative stress, and cellular injury in distant organs such as the kidneys (9). The systemic inflammation following cerebral injury activates immune cells and promotes the release of pro-inflammatory cytokines, which can directly damage renal vasculature and tubular cells. Additionally, ischemia-reperfusion injury—exacerbated by surgical intervention or aggressive blood pressure management—further compromises renal function through oxidative damage and mitochondrial dysfunction (10, 11). Despite growing understanding of these mechanisms, the inability to accurately monitor or predict renal injury in its early stages represents a significant gap in current neurocritical care.

Among potential biomarkers, lactate dehydrogenase (LDH) and albumin have shown promise in reflecting tissue injury and systemic inflammation—both of which are central to the pathogenesis of AKI (12). LDH, an enzyme released during cellular damage, serves as a sensitive marker of hypoxia and metabolic stress, while hypoalbuminemia, common in critically ill patients, reflects both nutritional status and the degree of systemic inflammation (13). The LAR combines these two parameters, capturing both cellular damage (via LDH) and inflammatory burden (via albumin), and has been shown to have prognostic value in various conditions such as cancer, cardiovascular disease, and liver cirrhosis (14, 15). However, its potential utility in predicting AKI in ICH patients remains unexplored.

Given the multifactorial and dynamic nature of AKI in ICH, the LAR may provide a novel and easily accessible biomarker that reflects the dual pathophysiological processes of tissue injury and systemic inflammation. Early identification of at-risk patients using this ratio could facilitate proactive management, potentially mitigating renal injury and improving overall outcomes.

Therefore, this study aims to explore the association between the LAR and AKI in patients with ICH, with the hypothesis that this ratio could serve as an effective early predictor of renal dysfunction. The findings could offer valuable insights into AKI pathogenesis in ICH and address the current clinical need for predictive tools in this critically ill population.

## Methods

### Study design

This retrospective, multicenter cohort study analyzed data from 6,465 patients diagnosed with ICH across three medical centers. Patient records were collected from West China Hospital of Sichuan University (spanning December 2010 to August 2019), The First People's Hospital of Longquan Yi District, Chengdu (from December 2016 to November 2020), and the Affiliated Hospital of Chengdu University (between August 2012 and November 2020). Ethical approval for this study was granted by the institutional review boards of all participating hospitals. Given the nature of the research as a clinical audit, the requirement for informed consent was waived. The study was conducted in accordance with the STROBE reporting guidelines and complied with the ethical principles set forth in the Declaration of Helsinki (1964) and its subsequent amendments.

### Patient selection

The study enrolled all patients diagnosed with ICH. Confirmation of ICH was established upon admission via computed tomography or magnetic resonance imaging, or through intraoperative assessment by a neurosurgeon during hospitalization. Individuals younger than 18 years were excluded. Additional exclusion criteria included: (1) secondary causes of hemorrhage, such as ischemic stroke with hemorrhagic conversion, traumatic brain injury, cerebral aneurysms, arteriovenous malformations, coagulopathy-related hemorrhage, and other non-primary ICH etiologies; (2) pre-existing acute kidney injury (AKI) prior to hospital admission; and (3) absence of serum LAR measurements within the first 24 h of admission.

### Data collection

We gathered comprehensive demographic and clinical data, including variables such as age, sex, smoking and alcohol use history, hypertension, diabetes mellitus, hematoma site, hematoma size, and whether intraventricular hemorrhage was present. The severity of ICH upon admission was evaluated using the Glasgow Coma Scale (GCS), alongside documentation of treatment modalities. Laboratory parameters used in this study—including neutrophil count, white blood cell count, lymphocyte count, monocyte count, glucose, creatinine, albumin, globulin, total cholesterol, high-density lipoprotein, and LDH—were obtained from the first blood draw within 24 h of admission. The LAR was subsequently calculated based on these values. Receiver operating characteristic (ROC) curve analysis was employed to determine the optimal cutoff value of LAR for discriminating between different clinical outcomes. Further statistical analyses were performed to assess the relationships between clinical features, laboratory markers, and patient outcomes. Missing data: we addressed missing baseline covariates using multiple imputation by chained equations (MICE), assuming missing at random. The imputation model included all variables used in the multivariable analyses to preserve associations among covariates and the outcome.

Venous blood samples were collected within the first 24 h of hospital admission, prior to any major interventions. Serum lactate dehydrogenase (LDH) was measured using an enzymatic colorimetric method based on the oxidation of L-lactate to pyruvate with concomitant reduction of NAD^+^ to NADH, quantified spectrophotometrically at 340 nm. Serum albumin concentration was determined using the bromocresol green dye-binding method, with absorbance measured at 628 nm. All assays were performed in the central laboratories of the participating hospitals using automated biochemical analyzers (Hitachi 7600 series, Hitachi High-Technologies, Tokyo, Japan, or equivalent). Daily two-point calibration and participation in external quality-assessment programs ensured analytical accuracy. The LAR was calculated as LDH (U/L) divided by serum albumin (g/L), both obtained from the same blood draw. Other biochemical parameters, including serum creatinine, glucose, and lipid profile, were determined according to standard hospital protocols using automated analyzers, with quality control performed according to Clinical and Laboratory Standards Institute (CLSI) guidelines.

### Outcomes

The primary outcome measure was the occurrence of AKI. AKI was defined in accordance with the kidney disease: Improving Global Outcomes (KDIGO) Clinical Practice Guidelines. Specifically, AKI was diagnosed if any of the following criteria were met: (1) an increase in serum creatinine by ≥0.3 mg/dl (≥26.5 μmol/L) within 48 h; (2) an increase in serum creatinine to ≥1.5 times the baseline value, which is known or presumed to have occurred within the prior 7 days; or (3) a reduction in urine output to < 0.5 ml/kg/h for more than six consecutive hours. In this study, baseline renal function was defined as the serum creatinine level on admission in patients without known pre-existing renal impairment. For patients with multiple creatinine measurements within the specified time frame, the highest value was used to determine AKI status.

### Statistical analysis

Continuous variables, such as age, GCS score, hematoma volume, leukocyte count, lymphocyte count, albumin concentration, platelet count, and glucose levels, were summarized as mean ± standard deviation (SD). The student's *t*-test was applied for normally distributed continuous variables, while the Mann–Whitney *U* test was employed for non-normally distributed data. Categorical variables were described as frequencies and percentages, with group comparisons conducted using either the chi-square test or Fisher's exact test, as appropriate.

Based on existing literature and clinical judgment, all relevant demographic information, baseline clinical variables, and laboratory parameters were considered for inclusion in logistic regression models. Univariate logistic regression analyses were initially performed to explore the relationship between each individual factor and the occurrence of AKI. Variables yielding *p*-values below 0.05 in univariate analyses were subsequently included in the multivariate logistic regression to adjust for potential confounding effects.

The discriminative ability of albumin, LDH, and the LAR for predicting AKI was evaluated using receiver-operating-characteristic curve (ROC) analysis, with the area under the ROC curve (AUROC) calculated to quantify predictive performance.

Additionally, subgroup analyses were performed to assess potential variations in outcomes across different strata, including age groups ( ≤ 65 vs. >65 years), sex, smoking status, alcohol consumption, hypertension, diabetes mellitus, hematoma location (infratentorial vs. supratentorial), hematoma volume ( ≤ 30 vs. >30 ml), presence of intraventricular hemorrhage, and GCS categories. These variables were selected based on clinical relevance, prior studies, and established risk scoring systems.

All statistical analyses were conducted using R software (version 4.2.1; R Foundation for Statistical Computing, Vienna, Austria). A two-tailed *p*-value < 0.05 was considered indicative of statistical significance.

## Results

**Figure 1:**
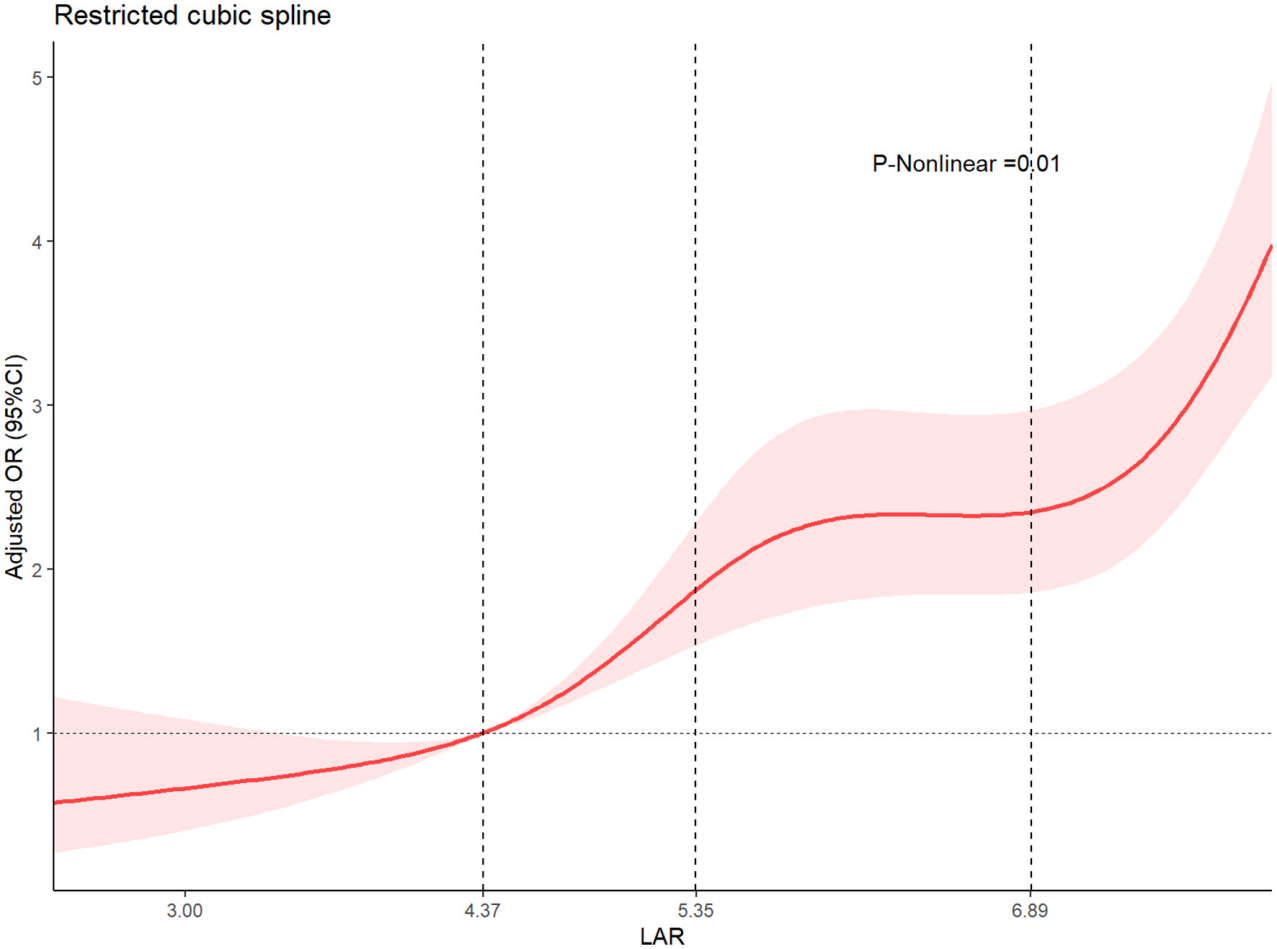
The receiver operating characteristic curves illustrating the predictive.

We screened 10,557 adults with ICH. After eligibility assessment, patients were excluded for: non-primary ICH etiologies (*n* = 95), missing LDH measurement (*n* = 4,051), and missing albumin measurement (*n* = 41). The final analytic sample comprised 6,465 patients (Figure 1).

Baseline demographic and clinical characteristics are summarized in Table 1. Among 6,465 patients with intracerebral hemorrhage (ICH), higher LAR quartiles were associated with older age (54.19 ± 14.91 years in Q1 vs. 69.73 ± 15.24 years in Q4; *p* < 0.001) and a greater proportion of females (30.2 vs. 53.3%; *p* < 0.001). Hypertension prevalence differed across LAR quartiles (Q1 61.6%, Q2 67.9%, Q3 66.2%, Q4 62.7%; *p* < 0.001), and intraventricular hemorrhage ranged from 17.2 to 31.0% (*p* < 0.001). There were no significant differences in smoking status, alcohol use, diabetes, or infratentorial location (*p* > 0.05). Hematoma volume was larger in Q4 than Q1 (27.84 ± 27.74 ml vs. 17.42 ± 20.63 ml; *p* < 0.001), with lower admission GCS scores (8.88 ± 4.23 vs. 12.87 ± 3.21; *p* < 0.001). Laboratory data showed that patients in Q4 had higher glucose (8.60 ± 4.09 vs. 6.63 ± 3.25 mmol/L), neutrophil counts (24.36 ± 29.70 vs. 18.72 ± 26.25 × 10^9^/L), white blood cell counts (12.85 ± 19.40 vs. 8.78 ± 3.48 × 10^9^/L), and creatinine (134.97 ± 166.10 vs. 80.38 ± 51.86 μmol/L) compared with Q1 (all *p* < 0.001). Lymphocyte counts decreased with increasing LAR (1.02 ± 0.96 vs. 1.28 ± 0.59 × 10^9^/L; *p* < 0.001). Table 2 shows that LAR was significantly associated with AKI risk in both unadjusted and adjusted models. When modeled as a continuous variable, each unit increase in LAR was associated with a 7% higher odds of AKI before adjustment (OR = 1.07, 95% CI 1.06–1.09, *p* < 0.001) and a 4% higher odds after adjustment (OR = 1.04, 95% CI 1.03–1.06, *p* < 0.001). In the dichotomized analysis, patients with LAR >5.61 had markedly higher AKI incidence (26.3%) compared with those with LAR ≤ 5.60 (9.6%), with unadjusted and adjusted ORs of 3.35 (95% CI 2.92–3.85) and 2.51 (95% CI 2.13–2.96), respectively (both *p* < 0.001). Quartile analysis revealed a dose–response relationship: compared with Q1 (LAR ≤ 4.36, AKI rate 6.0%), the odds of AKI progressively increased across quartiles—Q2 (4.37–5.35, 12.8% incidence; adjusted OR = 1.95, 95% CI 1.47–2.58), Q3 (5.36–6.89, 18.4%; adjusted OR = 2.62, 95% CI 1.99–3.44), and Q4 (>6.90, 30.9%; adjusted OR = 4.59, 95% CI 3.51–6.00), all with *p* < 0.001. This gradient supports a strong and independent association between higher LAR and increased AKI risk.

**Table 1:** Baseline characteristics of the patients with intracerebral hemorrhage.

**Characteristics**	**Lactic dehydrogenase to albumin ratio**					***p-*Value**	**Missing, *n* (%)**
	**Overall (*****n*** = **6,465)**	**Q1 (** ≤ **4.36**, ***n*** = **1,618)**	**Q2 (4.37–5.35**, ***n*** = **1,615)**	**Q3 (5.36–6.89**, ***n*** = **1,616)**	**Q4 (**>**6.90**, ***n*** = **1,616)**		
**Demographics**							
Age, years, mean (SD)	58.49 (14.74)	54.19 (14.61)	59.69 (13.91)	60.31 (14.34)	59.78 (15.24)	< 0.001	0 (0.00)
Female, *n* (%)	2,185 (33.8)	489 (30.2)	536 (33.2)	589 (36.4)	571 (35.3)	0.001	0 (0.00)
Smoking, *n* (%)						0.414	0 (0.00)
Never	4,301 (66.5)	1,038 (64.2)	1,082 (67.0)	1,098 (67.9)	1,083 (67.0)		
Current	1,766 (27.3)	471 (29.1)	438 (27.1)	423 (26.2)	434 (26.9)		
Ever	398 (6.2)	109 (6.7)	95 (5.9)	95 (5.9)	99 (6.1)		
Alcohol, *n* (%)	2,102 (32.5)	555 (34.3)	509 (31.5)	516 (31.9)	522 (32.3)	0.338	0 (0.00)
**Medical history**, ***n*** **(%)**							
Hypertension	4,175 (64.6)	996 (61.6)	1,097 (67.9)	1,069 (66.2)	1,013 (62.7)	< 0.001	0 (0.00)
Diabetes	678 (10.5)	160 (9.9)	166 (10.3)	155 (9.6)	197 (12.2)	0.07	0 (0.00)
**Hematoma characteristics**							
Hematoma intraventricular	1,622 (25.1)	278 (17.2)	370 (22.9)	473 (29.3)	501 (31.0)	< 0.001	0 (0.00)
Hematoma size (mean (SD))	22.06 (26.37)	17.42 (26.03)	19.69 (23.42)	23.82 (27.17)	27.84 (27.74)	< 0.001	1,305 (20.19)
Hematoma infratentorial	1,167 (18.1)	293 (18.1)	276 (17.1)	295 (18.3)	303 (18.8)	0.661	0 (0.00)
GCS (mean (SD))	10.99 (4.07)	12.87 (3.21)	11.67 (3.70)	10.54 (3.96)	8.88 (4.23)	< 0.001	30 (0.46)
**Laboratory tests**							
Glucose, mmol/L	7.56 (3.25)	6.63 (2.35)	7.38 (3.00)	7.65 (3.05)	8.60 (4.08)	< 0.001	119 (1.84)
Neutrophil, 10^9^/L	21.17 (27.91)	18.72 (26.52)	18.56 (25.64)	23.04 (29.08)	24.36 (29.73)	< 0.001	336 (5.20)
White blood cell count, 10^9^/L	10.49 (10.41)	8.78 (3.48)	9.67 (3.90)	10.66 (4.66)	12.85 (19.40)	< 0.001	330 (5.10)
Lymphocyte, 10^9^/L	1.11 (0.73)	1.28 (0.59)	1.12 (0.70)	1.03 (0.59)	1.02 (0.96)	< 0.001	336 (5.20)
Globulin, g/L	26.55 (5.10)	25.77 (4.46)	26.46 (4.57)	27.07 (5.12)	26.92 (6.02)	< 0.001	106 (1.64)
Monocyte, 10^9^/L	0.57 (3.10)	0.46 (0.23)	0.50 (0.29)	0.57 (0.69)	0.77 (6.18)	0.028	353 (5.46)
Creatinine, μmol/L	101.54 (125.47)	80.38 (81.86)	93.82 (122.91)	96.96 (109.29)	134.97 (166.10)	< 0.001	7 (0.11)

GCS, Glasgow Coma Scale; SD, standard deviation; Missing, n (%), refers to missingness in the raw dataset. Variables with missingness were imputed using MICE for multivariable analyses; only LDH and albumin missingness led to exclusion because LAR could not be calculated.

**Table 2:** Unadjusted and adjusted associations between lactic dehydrogenase to albumin ratio and acute kidney injury.

**LAR Categories**	**LAR(%)**	**Events, *n* (%)**	**Logistic regression Unadjusted OR**	***p-*Value**	**Logistic regression Adjusted OR**	***p-*Value**
Continuous	per 1	NA	1.07 (1.06–1.09)	< 0.001	1.04 (1.03–1.06)	< 0.001
Dichotomy	≤ 5.60	347/3,606 (9.6%)	1 (reference)	1 (reference)	1 (reference)	1 (reference)
	>5.61	752/2,859 (26.3%)	3.35 (2.92–3.85)	< 0.001	2.51 (2.13–2.96)	< 0.001
**Quartile**						
Q1	≤ 4.36	97/1,618 (6%)	1 (reference)	1 (reference)	1 (reference)	1 (reference)
Q2	4.37–5.35	206/1,615 (12.8%)	2.29 (1.78–2.95)	< 0.001	1.95 (1.47–2.58)	< 0.001
Q3	5.36–6.89	297/1,616 (18.4%)	3.53 (2.78–4.49)	< 0.001	2.62 (1.99–3.44)	< 0.001
Q4	>6.90	499/1,616 (30.9%)	7.00 (5.56–8.82)	< 0.001	4.59 (3.51–6.00)	< 0.001

Cl, confidence interval; NA, not available; OR, odds ratio.

**Figure 2:**
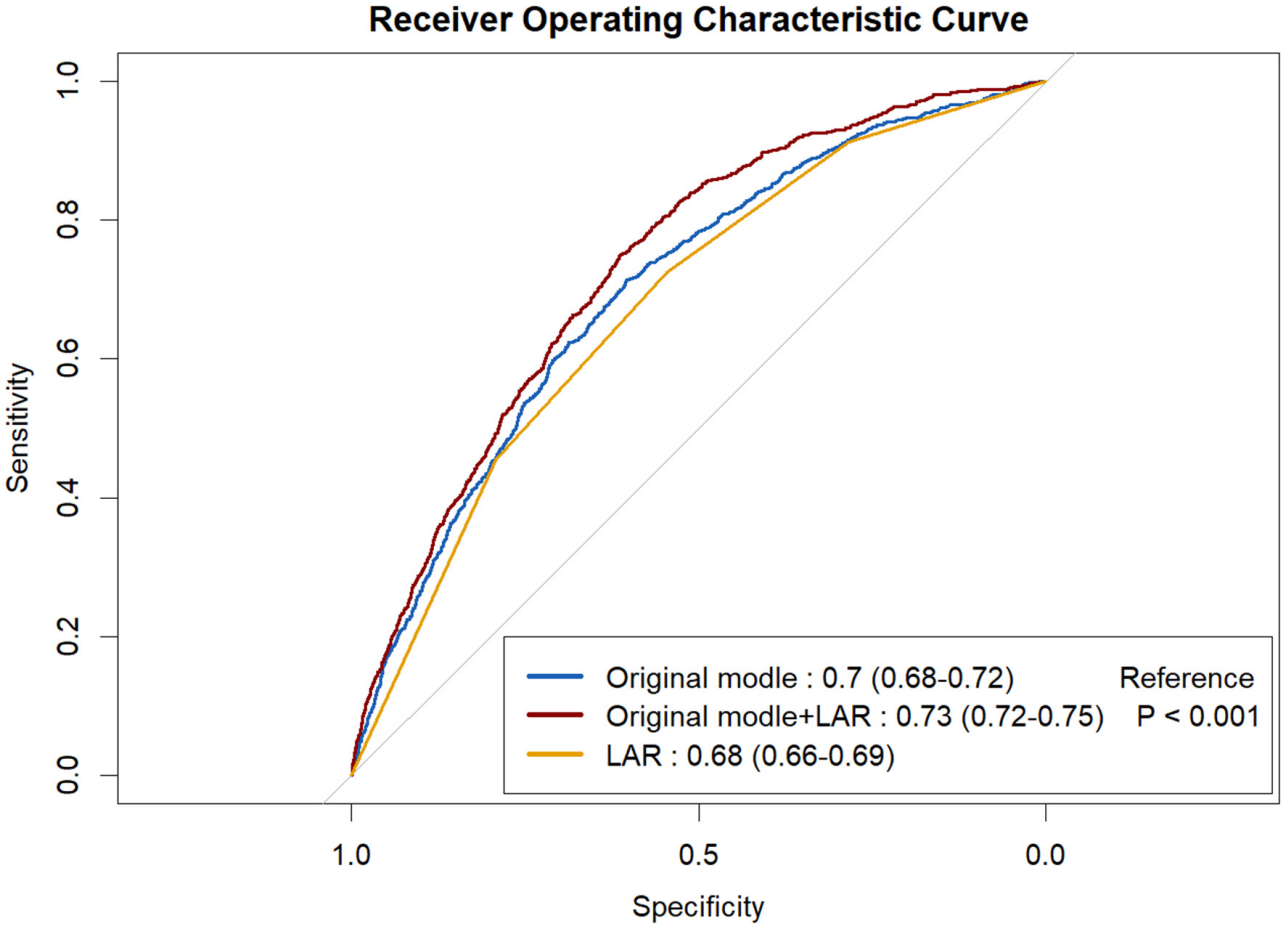
The restricted cubic spline depicting lactic dehydrogenase-to-albumin ratio Associated with acute kidney injury among patients with intracerebral hemorrhage.

**Figure 3:**
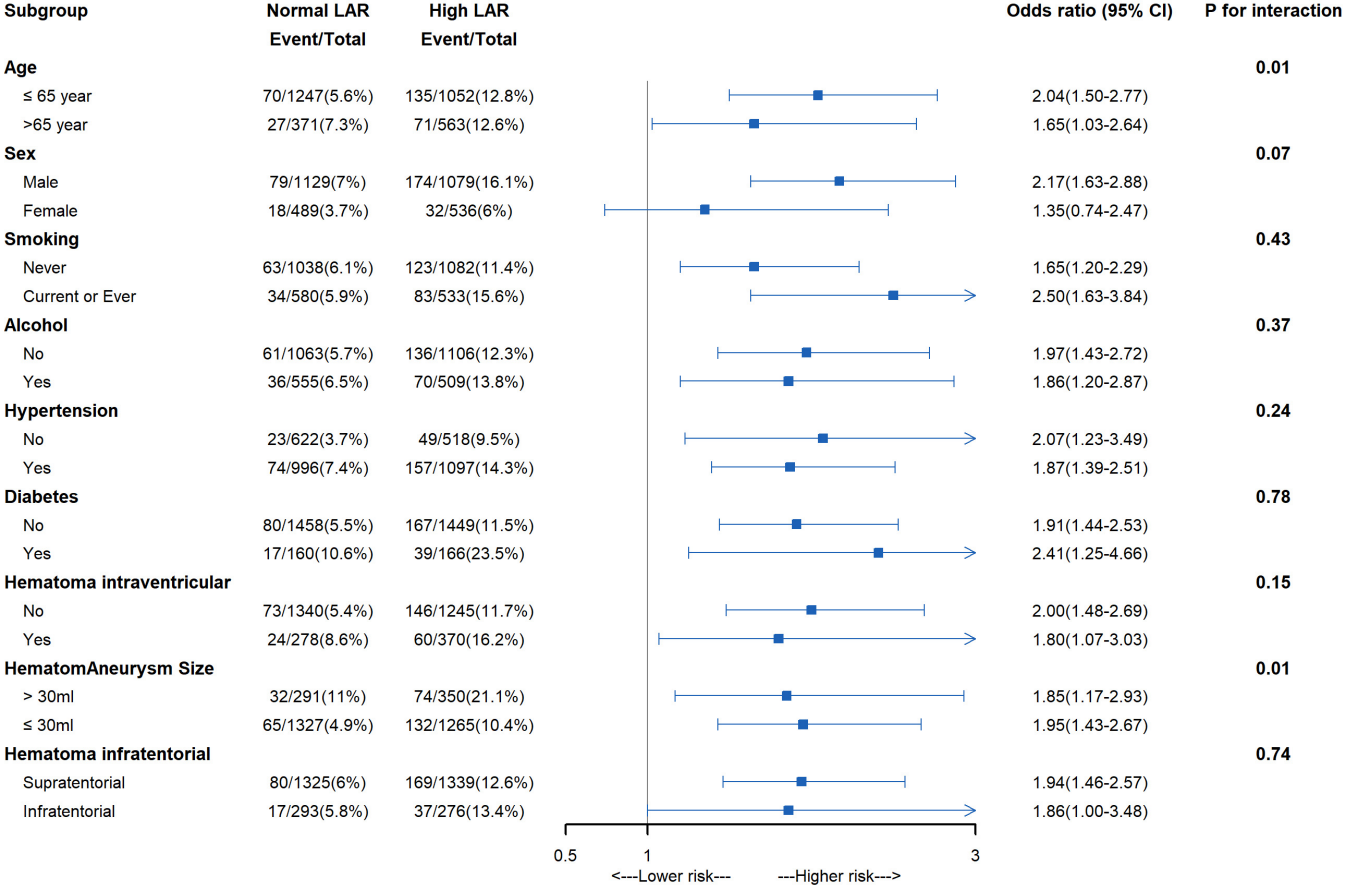
Subgroup analysis of association between lactic dehydrogenase-to-albumin ratio to acute kidney injury.

The adjusted restricted cubic spline curve demonstrated a significant non-linear relationship between lactic dehydrogenase-to-albumin ratio (LAR) and the risk of acute kidney injury (AKI) in patients with intracerebral hemorrhage. At lower LAR values, the adjusted odds ratio for AKI remained close to 1, indicating minimal risk elevation. However, the risk began to rise steadily once LAR exceeded approximately 4.37, plateaued slightly between 5.35 and 6.89, and increased sharply thereafter, with the highest risk observed beyond 6.89. The 95% confidence interval (shaded area) confirmed the precision of these estimates, and the *p*-value for non-linearity was statistically significant (*p* = 0.01), indicating that the association was not purely linear but characterized by threshold effects and accelerated risk escalation at higher LAR levels (Figure 2). In subgroup analyses, elevated LAR was consistently associated with a higher risk of a AKI across most clinical categories. The association appeared stronger in patients aged ≤ 65 years (OR = 2.04, 95% CI 1.50–2.77) than in those >65 years (OR = 1.65, 95% CI 1.03–2.64; *p* for interaction = 0.01). The effect was also more pronounced in patients with hematoma volume >30 ml (OR = 1.85, 95% CI 1.17–2.93) compared with those ≤ 30 ml (OR = 1.95, 95% CI 1.43–2.67; *p* for interaction = 0.01). No statistically significant interactions were observed for sex, smoking status, alcohol use, hypertension, diabetes, presence of intraventricular hemorrhage, or hematoma location (*p* for interaction >0.05 for all). Overall, the findings indicate that the adverse impact of high LAR on AKI risk is broadly consistent across diverse patient subgroups, with notable heterogeneity by age and hematoma size (Figure 3).

**Figure 4:**
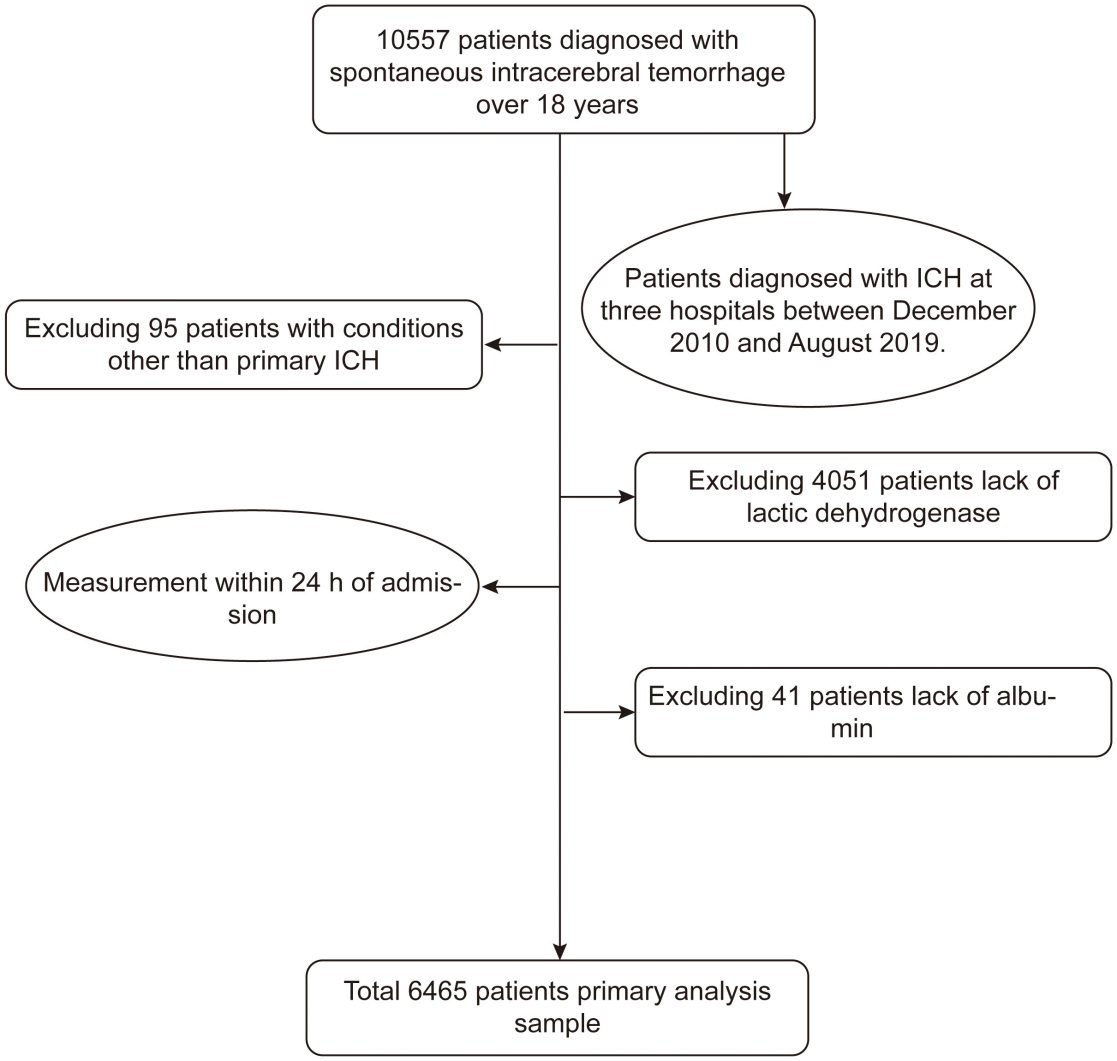
The receiver operating characteristic curves illustrating the predictive value of the LAR, original model, and original model+ LAR for AKI.

When evaluated using ROC curve analysis, the discriminative ability of LAR alone for predicting acute kidney injury was moderate, with an AUC of 0.68 (95% CI, 0.66–0.69). The original multivariable model without LAR achieved an AUC of 0.70 (95% CI, 0.68–0.72). Incorporating LAR into the original model significantly enhanced predictive performance, yielding an AUC of 0.73 (95% CI, 0.72–0.75; *p* < 0.001), demonstrating the incremental prognostic value of LAR for AKI risk stratification (Figure 4). To further evaluate the comparative predictive value of LAR, LDH, and albumin, we performed additional ROC curve analyses. As shown in Supplementary Figure S1, LAR demonstrated superior discriminative ability (AUC 0.69) compared with LDH (AUC 0.64) and albumin (AUC 0.65).

## Discussion

In this multicenter study, we explored the role of the lactic dehydrogenase to albumin ratio as a predictor of AKI in patients with ICH. Our results indicate that elevated LAR is strongly associated with AKI risk and shows a dose-response relationship between higher LAR levels and increased AKI likelihood; however, given the retrospective design, a causal relationship cannot be inferred. These findings suggest that LAR could enhance predictive models for AKI in ICH, allowing for earlier identification and more targeted management.

This study highlights LAR as a composite biomarker integrating markers of cellular injury (LDH) and systemic inflammation (ALB) that is associated with AKI risk rather than implying causation in patients with ICH (16, 17). Mechanistically, elevated LDH in the circulation reflects cytolytic release from hypoxic or injured tissues and heightened glycolytic flux; in the kidney, ischemia–reperfusion and systemic inflammation injure tubular epithelium, disrupt mitochondrial function, trigger DAMP-mediated innate immunity, oxidative stress, and endothelial dysfunction. SDH-driven metabolic reprogramming fuels inflammatory macrophage responses, while lactate–LDH pathways modulate immune activation and microvascular injury (18–20). While LDH and ALB have been individually associated with prognosis in stroke and critical illness, ICH-specific evidence on the combined index is limited; however, LAR has shown prognostic associations in related acute conditions (e.g., stroke-associated pneumonia; severe infection/ICU cohorts) (21–23). Our findings demonstrate that elevated LAR is strongly associated with AKI risk, with patients exhibiting LAR values above 5.61 showing significantly increased odds of renal dysfunction. The stepwise rise in AKI risk across LAR quartiles further supports its role as a dose-dependent risk indicator.

The mechanistic plausibility lies in the interaction between systemic inflammation and renal injury in ICH. From the albumin perspective, hypoalbuminemia mirrors systemic inflammation and capillary leak and reduces oncotic pressure and antioxidant/ligand-carrying capacity, favoring interstitial edema, glycocalyx shedding, and impaired renal perfusion; these processes synergize with LDH-reflected tissue injury to raise LAR (21). The LAR thus condenses these processes into a single, clinically practical marker (22, 23). Our data suggest that higher LAR levels correlate with a heightened inflammatory burden, which may underlie the increased susceptibility to AKI in critically ill ICH patients.

Beyond the dose–response association, patient-level patterns show that LAR elevation often precedes measurable renal impairment, consistent with its role as an upstream risk signal rather than a consequence of reduced kidney function. When modeled continuously, dichotomized at the Youden cut-off (~5.61), or stratified into quartiles, LAR retained an independent association with AKI after multivariable adjustment. The steeper slope in the mid-to-high range is compatible with threshold phenomena (e.g., tubular transport collapse, microvascular dysregulation) once inflammatory–metabolic stress surpasses renal compensatory capacity (16, 17).

### Effect modification by age

The association was stronger in younger patients ( ≤ 65 years). A likely explanation is that fewer chronic comorbidities (malnutrition/inflammation) in younger individuals mean that acute LDH/ALB shifts more specifically reflect the current inflammatory–ischemic insult, improving the signal-to-noise ratio for LAR; conversely, in older or large-hematoma patients, multiple concurrent pathways (hemodynamic instability, multiorgan ischemia) may dilute LAR's incremental value (“risk saturation”). This interpretation is consistent with literature describing kidney–brain vulnerability and hemodynamic contributors to post-stroke AKI, and highlights the need to compare LAR with kidney-specific biomarkers (e.g., proenkephalin) in ICH cohorts (24–26).

Importantly, integrating LAR into a base clinical model yielded a statistically and clinically meaningful gain in predictive discrimination (AUC 0.70 to 0.73), with potential for earlier identification of high-risk patients and timely deployment of reno-protective measures. Given that LAR is inexpensive, rapidly measurable, and universally available, it is particularly attractive for acute care settings and resource-limited environments. These correlations between patient phenotypes, biochemical profiles, and AKI risk provide a compelling rationale for incorporating LAR into ICH risk stratification frameworks, while also highlighting the need for prospective validation and exploration of intervention thresholds.

### Comparison with prior studies

Our findings align with, and extend, prior ICH research on AKI. Observational and trial-based analyses have shown that more intensive or rapid systolic BP reduction after ICH associates with increased AKI risk and worse renal safety signals, including within ATACH-2 and related cohorts (25, 27, 28). Medication-specific analyses have further suggested that certain antihypertensives may be linked to higher AKI rates in this setting (29). Beyond hemodynamics, ICH cohorts have identified classical risk factors—such as higher mannitol infusion rates, older age, larger hematomas, and baseline comorbidity burden—as contributors to AKI or renal failure (30, 31). Recent mini-reviews summarize additional contributors and emphasize the prognostic impact of AKI after ICH (32). Against this backdrop, our study adds a biochemical composite perspective: LAR integrates tissue-injury and hypoalbuminemia/inflammation signals and remains independently associated with AKI after adjustment for ICH severity. While ICH-specific LAR reports are scarce, convergent evidence from sepsis/ICU cohorts shows higher LAR predicts incident AKI, RRT use, and mortality, supporting the generalizability of injury–inflammation composites as early kidney-risk indicators (33). Finally, contemporary reviews of AKI biomarkers highlight the limitations of creatinine/urine output and the value of adding damage/stress markers; our LAR findings complement these frameworks by offering a low-cost, widely available composite for risk stratification in ICH (26).

Despite the strengths of our multicenter design and large sample size, this study has several important limitations. First, the retrospective design inherently limits the ability to establish causal relationships between LAR and AKI, and residual confounding from unmeasured factors cannot be excluded. In particular, systemic infection—a common complication in patients with intracerebral hemorrhage—can independently increase lactate dehydrogenase levels and decrease serum albumin, potentially confounding the observed association between LAR and AKI. Second, although LAR is derived from routine laboratory parameters, variability in assay methods across participating institutions could introduce measurement bias. Third, all participating centers were located in China, and the lack of external validation in other healthcare systems may limit generalizability. Fourth, the study focused only on short-term outcomes without evaluating long-term renal function or post-discharge survival. Fifth, the study did not differentiate the extent to which AKI directly contributed to mortality in patients who also had ICH. In-hospital deaths were attributable to ICH, AKI, or both, but the overlapping nature of these conditions made attribution challenging. Future studies with cause-specific mortality analyses are warranted to clarify the independent impact of AKI on outcomes after ICH. Prospective, multicenter studies with standardized laboratory protocols, external validation across diverse populations, and longitudinal follow-up are needed to determine whether early AKI risk stratification via LAR can improve long-term renal and overall outcomes.

#### Timing of AKI

Precise onset timestamps were not uniformly available, precluding the calculation of the median time to AKI and formal time-to-event or landmark analyses. Although LAR was anchored to admission laboratories ( ≤ 24 h), the absence of onset timing precludes definitive claims of early prediction. Consequently, LAR should be interpreted as a baseline risk-stratification measure; future prospective studies with standardized time-stamped capture are warranted to establish temporal precedence. Renal recovery was not evaluated: serial post-AKI creatinine measurements and post-discharge follow-up necessary to define recovery (e.g., MAKE30/MAKE90, dialysis dependence) were not uniformly available across centers. In conclusion, LAR emerges as a promising, accessible biomarker for early identification of AKI risk in ICH patients. Its ability to integrate inflammation and tissue injury markers could help refine risk stratification and guide early clinical interventions in neurocritical care.

## Conclusions

This study highlights the potential of LAR as a biomarker associated with AKI risk in patients with ICH. LAR combines markers of cellular injury and inflammation, making it a valuable tool for early risk stratification; nevertheless, causality cannot be established owing to the retrospective design. Incorporating LAR into predictive models improves accuracy, offering a simple and cost-effective method for identifying high-risk patients. Future studies with longer follow-up and validation are needed to confirm its clinical utility and impact on patient outcomes.

## Data Availability

The original contributions presented in the study are included in the article/supplementary material, further inquiries can be directed to the corresponding author.
