# Supplementary material for: The lactic dehydrogenase-to-albumin ratio predicts acute kidney injury in patients with intracerebral hemorrhage: a multicenter cohort study
Source: Front Neurol. 2025 Dec 18;16:1606881. doi: 10.3389/fneur.2025.1606881 (PMC12756156; doi:10.3389/fneur.2025.1606881)

Appendix

Supplementary Figure 1: Receiver operating characteristic curves of lactic dehydrogenase to albumin ratio, albumin, and LDH for predicting acute kidney injury in patients with intracerebral hemorrhage.
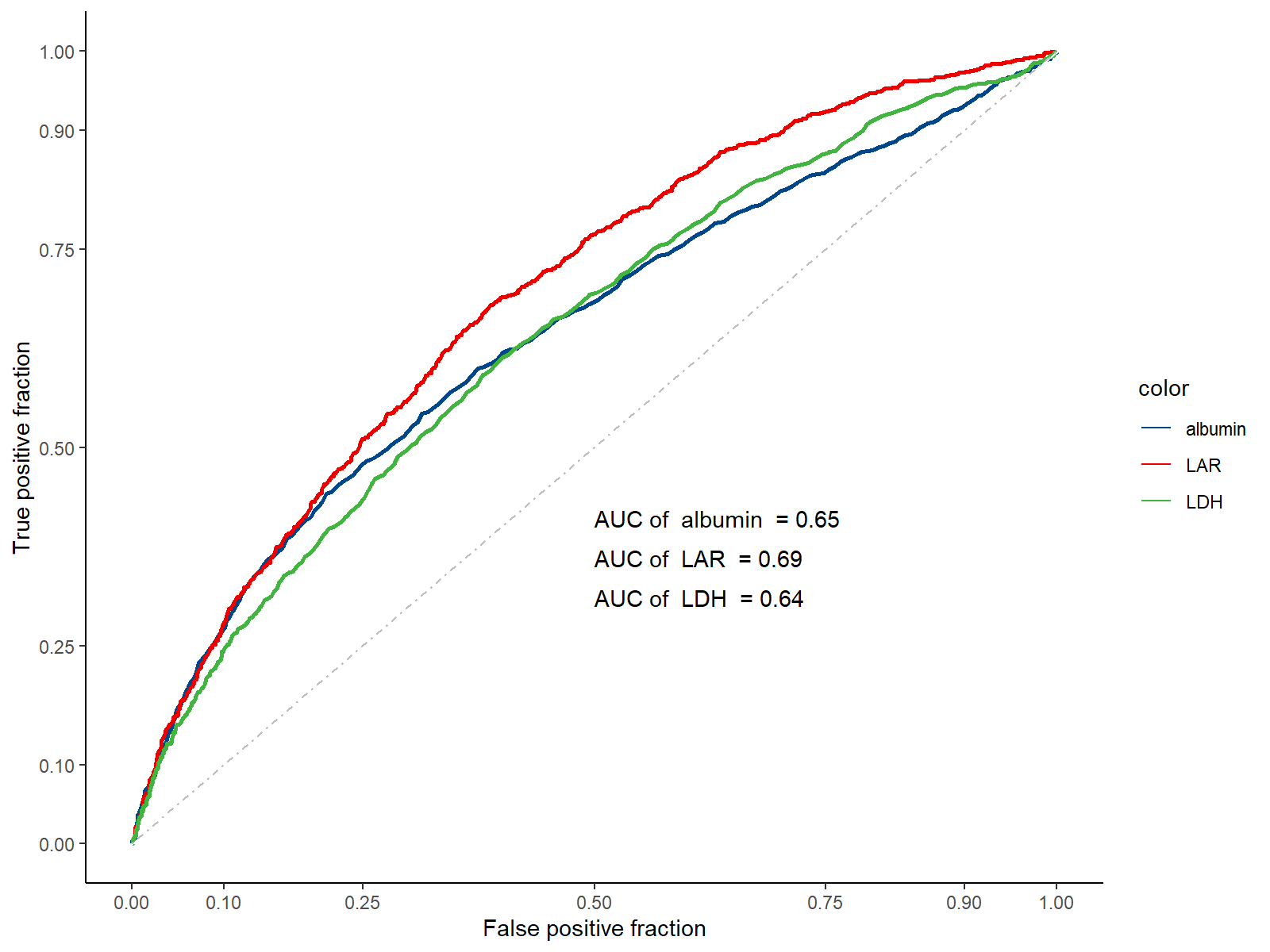

Supplement: Supplementary file 1 [file Supplementary_file_1.docx]
